# Predicting the distributions of predator (snow leopard) and prey (blue sheep) under climate change in the Himalaya

**DOI:** 10.1002/ece3.2196

**Published:** 2016-05-18

**Authors:** Achyut Aryal, Uttam Babu Shrestha, Weihong Ji, Som B. Ale, Sujata Shrestha, Tenzing Ingty, Tek Maraseni, Geoff Cockfield, David Raubenheimer

**Affiliations:** ^1^ Institute of Natural and Mathematical SciencesMassey UniversityPrivate Bag 102904AucklandNew Zealand; ^2^ School of Life and Environmental SciencesFaculty of ScienceThe University of SydneySydneyNew South WalesAustralia; ^3^ Institute for Agriculture and the EnvironmentUniversity of Southern QueenslandToowoombaQueenslandAustralia; ^4^ Biological SciencesUniversity of IllinoisChicagoIllinois60607; ^5^ Snow Leopard Conservancy18030 Comstock AvenueSonomaCalifornia95476; ^6^ Department of Biological ScienceUniversity of Massachusetts BostonBostonMassachusetts; ^7^ School of CommerceUniversity of Southern QueenslandToowoombaQueenslandAustralia; ^8^ The Charles Perkins Centre and Faculty of Veterinary Science and School of Biological SciencesThe University of SydneySydneyNew South WalesAustralia

**Keywords:** Climate change, distribution, impact, niche, prey–predator interaction

## Abstract

Future climate change is likely to affect distributions of species, disrupt biotic interactions, and cause spatial incongruity of predator–prey habitats. Understanding the impacts of future climate change on species distribution will help in the formulation of conservation policies to reduce the risks of future biodiversity losses. Using a species distribution modeling approach by MaxEnt, we modeled current and future distributions of snow leopard (*Panthera uncia*) and its common prey, blue sheep (*Pseudois nayaur*), and observed the changes in niche overlap in the Nepal Himalaya. Annual mean temperature is the major climatic factor responsible for the snow leopard and blue sheep distributions in the energy‐deficient environments of high altitudes. Currently, about 15.32% and 15.93% area of the Nepal Himalaya are suitable for snow leopard and blue sheep habitats, respectively. The bioclimatic models show that the current suitable habitats of both snow leopard and blue sheep will be reduced under future climate change. The predicted suitable habitat of the snow leopard is decreased when blue sheep habitats is incorporated in the model. Our climate‐only model shows that only 11.64% (17,190 km^2^) area of Nepal is suitable for the snow leopard under current climate and the suitable habitat reduces to 5,435 km^2^ (reduced by 24.02%) after incorporating the predicted distribution of blue sheep. The predicted distribution of snow leopard reduces by 14.57% in 2030 and by 21.57% in 2050 when the predicted distribution of blue sheep is included as compared to 1.98% reduction in 2030 and 3.80% reduction in 2050 based on the climate‐only model. It is predicted that future climate may alter the predator–prey spatial interaction inducing a lower degree of overlap and a higher degree of mismatch between snow leopard and blue sheep niches. This suggests increased energetic costs of finding preferred prey for snow leopards – a species already facing energetic constraints due to the limited dietary resources in its alpine habitat. Our findings provide valuable information for extension of protected areas in future.

## Introduction

Climate change is recognized as one of the biggest challenges to biodiversity worldwide (Garcia et al. 2014). It has already affected species distribution, community composition (Parmesan and Yohe 2003), and the population dynamics of predator–prey species (Gilg et al. 2009) and caused spatial incongruity of predator–prey habitats (Schweiger et al. 2012). Future climate change is likely to impact species demography and distribution and disrupt biotic interactions (Garcia et al. 2014). Understanding this is particularly useful in the formulation of conservation policy, adaptation planning, and assessing the extent of vulnerability and reducing the risk of future biodiversity losses (Nazeri et al. 2012, 2014; Kujala et al. 2013; Shrestha and Bawa 2014).

The Himalayan region is considered to be one of the most vulnerable regions to climate change (Shrestha et al. 2012). Impacts have been seen in glaciers, hydrology, agriculture, biodiversity, ecosystems, human health, and livelihoods (Xu et al. 2009). Studies, albeit limited and localized, have shown that climate change has shifted altitudinal ranges of plants (Telwala et al. 2013) and changed the distribution and breeding behaviors of birds, reptiles, amphibians, and butterflies (Acharya and Chettri 2012) in the Himalayan region. It is predicted that current suitable habitats of species (Kumar 2012; Shrestha and Bawa 2014) will be further altered.

The endangered snow leopard (*Panthera uncia*) inhabiting the rugged and fragile landscape of the Himalaya (Jackson and Ahlborn 1984) is one of the large predators in the energy‐deficient environments of high altitudes. Therefore, they are critical for maintaining ecosystem process, function, and resilience (Ripple et al. 2014). Furthermore, understanding the current and future distributions of snow leopard and its one of the major preys, blue sheep (*Pseudois nayaur*), is important not only for their protection but also for maintaining the health of mountain ecosystems where they reside (Lyngdoh et al. 2014).

Previous studies (e.g., Forrest et al. 2012) based on abiotic factors ignored biotic (prey) influences on the predicted changes on snow leopard distribution in the Himalayan region. Wegge et al. (2012) analyzed the diet of snow leopard from Manang, Nepal, and discovered that blue sheep was the most common diet (~92% of dietary composition). Therefore, the inclusion of biotic information including prey distribution in bioclimatic models will certainly improve current and future predictions of predator distribution and thereby also reduce uncertainty (Peers et al. 2014). While the distribution of snow leopard (predator) influences the distribution of blue sheep (prey) and vice versa (Aryal et al. 2013, 2014a), modeling snow leopard habitat together with that of its preferred, blue sheep, will produce a plausible prediction of current and future distributions. It should be noted that the dietary diversity of the snow leopard varies with the locations and the Himalayan region has the highest dietary diversity of snow leopard's preys ranging from Himalayan tahr (*Hemitragus jemlahicus*)*,* blue sheep, argali (*Ovis ammon*), serow (*Capricornis thar*), goral (*Naemorhedus goral*), musk dear (*Moschus* spp.), marmots (*Marmota* spp.), pikas (*Ochotona* spp.), large gallinaceous birds, and also domesticated animals (Lovari et al. 2009; Lyngdoh et al. 2014). However, blue sheep and Himalayan thar are the commonest prey species, the former one is the most represented prey species while later one is more abundant in Sagarmatha National Park of Nepal (Lovari et al. 2009, 2013).

Our objective in this study was to map current habitats of snow leopard and blue sheep and investigate the extent of niche overlap between them by including biotic interactions combined with environmental, bioclimatic, and topographic features and occurrence information in bioclimatic models. We then predicted changes in the distribution of snow leopard and blue sheep under future climate conditions to 2030 and 2050 and examined whether there was any current and future spatial matches and mismatches between the predicted distributions of snow leopard and blue sheep in Nepal Himalaya. Finally, we assessed the effectiveness of protected areas to encompass current distributions of snow leopard and blue sheep habitats and analyzed the relevance of protected areas vis‐à‐vis snow leopard distribution under climate change.

## Materials and Methods

### Study area and species

This study covers the entire area of snow leopard and blue sheep distribution in Nepal including all their presence protected areas and outside the protected areas of Nepal (for details about snow leopard distribution map of Nepal, see Aryal et al. 2014b; and for blue sheep, see Aryal et al. 2013). About 86% of Nepal is covered by hills and high mountains and rugged terrain covered by snow in winter (Bhuju et al. 2007) that offers ecological niche for the snow leopard and blue sheep. The Himalayan region of Nepal comprises 10% of the total habitat globally for snow leopard with an estimated population of 300–350 individuals (Aryal et al. 2013, 2014a). Snow leopards and blue sheep are normally found between 2500 and 5500 m altitude in alpine and subalpine grasslands. Blue sheep, also called “bharal” and “naur,” is a major prey species of the snow leopard distributed throughout the Himalayan region (Jackson and Ahlborn 1984; Wegge et al. 2012; Lyngdoh et al. 2014).

### Species occurrence data

The data on species occurrences were compiled from field surveys conducted at different times between 2007 and 2014 in various localities of Nepal. Presence points of snow leopards were collected using standard sampling techniques of scats collection, pugmarks and camera traps (Ale 2007; Ale and Brown 2009), and were later validated using molecular techniques (Aryal et al. 2014a,b,c). Presence points of blue sheep were collected by direct observation during field surveys carried out as part of other research projects (Aryal et al. 2010, 2013, 2014a,b,c). Presence localities of each species were binned into 1‐km^2^ grid cell by removing multiple presence points retaining only one presence point per grid cell. The remaining 364 presence points for snow leopard and 201 for blue sheep were used for modeling.

### Environmental variables

We used 19 bioclimatic data obtained from WorldClim (www.worldclim.org) (Hijmans et al. 2005), land cover data acquired from global land cover share (http://www.glcn.org) (Latham et al. 2014), and altitude from global multiresolution terrain elevation data 2010 (https://lta.cr.usgs.gov/GMTED2010) (Danielson and Gesch 2011). Slope, aspect, and ruggedness were calculated from the elevation layer in ArcGIS 10.3 (ESRI , Redlands, CA, USA). All raster layers were resampled to 30 arc‐sec (~1 km) resolution to correspond to the original resolution of the WorldClim data. Altogether, we used 19 bioclimatic, four topographic, and one environmental variable (see Table S1 in Appendix S1). We extracted each bioclimatic, topographic, and environmental variable corresponding to the occurrence location of each species to observe multicollinearity between those variables, dropped highly correlated variables (Pearson's correlation coefficient, *r* ^2^ ≥ 0.75), and included the remaining nine variables [annual mean temperature, mean diurnal range, isothermality, temperature seasonality, annual precipitation, aspect, slope, roughness, land use/land cover (LULC)] for snow leopard and eight variables (annual mean temperature, mean diurnal range, isothermality, temperature seasonality, aspect, slope, roughness, and LULC) for blue sheep in the final model. We used the feature dataset of World Database on Protected Areas (WDPA) as a boundary layer for protected areas including buffer zones of Nepal (http://www.protectedplanet.net).

We used MIROC5, the latest version of a global climate model (GCM), MIROC (Model for Interdisciplinary Research on Climate) (Watanabe et al. 2010) to predict distributions of snow leopard and blue sheep under future climate scenarios. MIROC5 is able to capture various observed features of future climate for the South Asian region very well (Mishra et al. 2014; Sharmila et al. 2015). We downloaded MIROC5 data for RCP4.5 scenarios for two different time periods (2030 and 2050) from www.ccafs-climate.org (Ramirez–Villegas and Jarvis 2010). We assume that RCP4.5 is a reasonable carbon emission scenario in which the total radiative forcing reaches 4.5 W/m^2^ (approximately 650 ppm CO_2_ equivalent) by the end of the 21st century and stabilizes thereafter due to the employment of a range of technologies and policies for reducing greenhouse gas emissions and radiative forcing (Thomson et al. 2011).

### Modeling

We used a maximum entropy (MaxEnt) species distribution model based on the principle of maximum entropy to model potential distribution of species (Phillips et al. 2006). It is the most widely used species distribution tool (Kramer‐Schadt et al. 2013) and is superior to other species distribution models in terms of performance (Elith et al. 2006; Wisz et al. 2008). Our data (presence only) fit best with MaxEnt as it uses presence‐only data and uses background environmental data of the study area. Nevertheless, MaxEnt has a few limitations that have been well discussed in the recent literature such as sampling bias of occurrence points, region used for background sampling, selection of features, and selection of regularization multiplier in the model (Elith et al. 2011; Kramer‐Schadt et al. 2013; Radosavljevic and Anderson 2014). The data collected from surveys during field trips may possess sampling biases, and sampling biases increase the spatial autocorrelation of localities (Boria et al. 2014) influencing the performance of the model and causing the model to overfit to the environmental biases (Reddy and Dávalos 2003; Phillips et al. 2009; Radosavljevic and Anderson 2014). We used two approaches to address the potential bias present in species occurrence records by applying a spatial filter (Kramer‐Schadt et al. 2013; Boria et al. 2014; Radosavljevic and Anderson 2014) and creating a bias grid to manipulate background selection while running the model (Kramer‐Schadt et al. 2013; Peers et al. 2014). Therefore, we ran MaxEnt models in three different scenarios: spatial filtering; bias grid; and base data (without spatial filtering and bias grid).

### Spatial filtering

Spatial filtering, which is performed by removing spatially autocorrelated points from the data, makes location data better for calibration and evaluation (Boria et al. 2014; Radosavljevic and Anderson 2014). We filtered localities with a minimum of 5 km distance and removed autocorrelated occurrence points located within 5 km of each other using SDMtoolbox, a python‐based GIS toolkit (Brown 2014). The spatial filter was limited to 5 km because of the high level of topographic heterogeneity in the study area (Anderson and Raza 2010; Boria et al. 2014). We used the remaining 172 and 136 spatially filtered occurrence points for snow leopards and blue sheep, respectively, for the modeling.

### Bias grid

We produced a bias grid to down‐weight highly clustered presence records from areas with highly intensive sampling (Elith et al. 2010; Peers et al. 2014). We created a Gaussian kernel density map of the occurrence locations using sampling bias distance of 100 km for snow leopard and 50 km for blue sheep to represent an approximate habitat range for each species. The density map was then rescaled to 1–20 value classes to prevent extreme down‐weighting of highly sampled cells (Elith et al. 2010; Peers et al. 2014).

We set the random test percentage to 25% (25% of presence points were assigned randomly to test the model performance), with 10 times cross‐validation and varying the values of the regularization multiplier. Warren and Seifert (2011) examined the effects of regularization on model performance and suggested evaluating the effects of regularization on model performance and structure. While change in regularization parameters substantially lowers overfitting of the model (Anderson and Gonzalez 2011; Radosavljevic and Anderson 2014), we manipulated regularization multiplier values (0.5, 1 (default), 1.5, 2, 3, 4, and 5) following the recommendations of Anderson and Gonzalez (2011), Radosavljevic and Anderson (2014), and Muscarella et al. (2014). We averaged the results of replicate runs from the models that were run in three different scenarios and with seven regularization multipliers. To avoid overfitting, we selected linear, quadratic, and hinge features (Phillips and Dudík 2008; Merow et al. 2013).

### Model evaluation

The most common model evaluation metrics are as follows: maximum training AUC (area under curve) value, maximum test AUC value (AUC_test_), minimum difference between training and test data (AUC_diff_), and information criteria such as Akaike information criteria (AIC) and Bayesian information criteria (BIC) (Muscarella et al. 2014). However, usage of the standard method of using receiver operating characteristic (ROC) curve or AUC value for model selection is not recommended in the literature (see Lobo et al. 2008; Peterson et al. 2008). Warren and Seifert (2011) also compared different evaluation methods and found that Akaike information criterion corrected for small sample sizes (AICc) outperformed all other AUC‐based methods. Furthermore, models selected based on AICc have lower omission rates and reduce overfitting (Muscarella et al. 2014). Therefore, we used the AICc based evaluation method for selecting the best performing MaxEnt model and designated the model with the lowest AICc values for further analysis. The statistical analysis was performed in R software.

### Niche overlap

To change the continuous value of the predicted distribution of MaxEnt into a binary (presence and absence) value, we used the equal training sensitivity and specificity threshold value as it provides the most accurate estimates (Cao et al. 2013). Assuming snow leopard can exist only in those areas where prey is available, we discounted snow leopard distributions derived from the models based on bioclimatic variables (climate‐only models) by incorporating the predicted distribution of blue sheep. We also calculated niche overlap between the predicted habitats of snow leopard and blue sheep using ENMTools (Warren et al. 2008) and compared the changes in niche overlap under future climate change scenarios. ENMTools measure niche overlap using *D* and *I* values calculated by comparing two normalized predicted distribution models produced by MaxEnt using estimated values of habitat suitability for each grid (Warren et al. 2010). The niche overlap between snow leopard and blue sheep is calculated using Schoener's *D* using the following formula.

$$D\left(p_{x},p_{y}\right)=1-\frac{1}{2}\sum_{n=i}\left|p_{x,i}-p_{y,i}\right|$$

where *p*_*x,i*_ and *p*_*y,i*_ represent the probability assigned by distribution model to grid cell *i* for species *x* and *y*, respectively. Schoener's *D* is typically applied with values of *p*_*xi*_ that reflect relative use of particular microhabitats and/or prey items and quantifies the degree of geographical overlap between two probability distributions with values ranges from 0 (distribution models have no overlap) to 1 (identical distribution models) (Warren et al. 2008). *I*‐statistic in ENMTools is based on Hellinger distance and measures the ability of the model to estimate true suitability of the habitat without biological assumptions to define the meaning of the *p*_*x,i*_ (Warren et al. 2008). *I*‐statistic is calculated as

$$I\left(p_{x},p_{y}\right)=1-\frac{1}{2}H\left(p_{x},p_{y}\right)$$

where *H* is the Hellinger distance and is defined as

$$H\left(p_{x},p_{y}\right)=\sqrt{\sum_{i}(\sqrt{p_{x,i}}-\sqrt{p_{y,i}})}$$

where *p_x_* and *p_y_* are as probability distributions.

## Results

**Figure 1:**
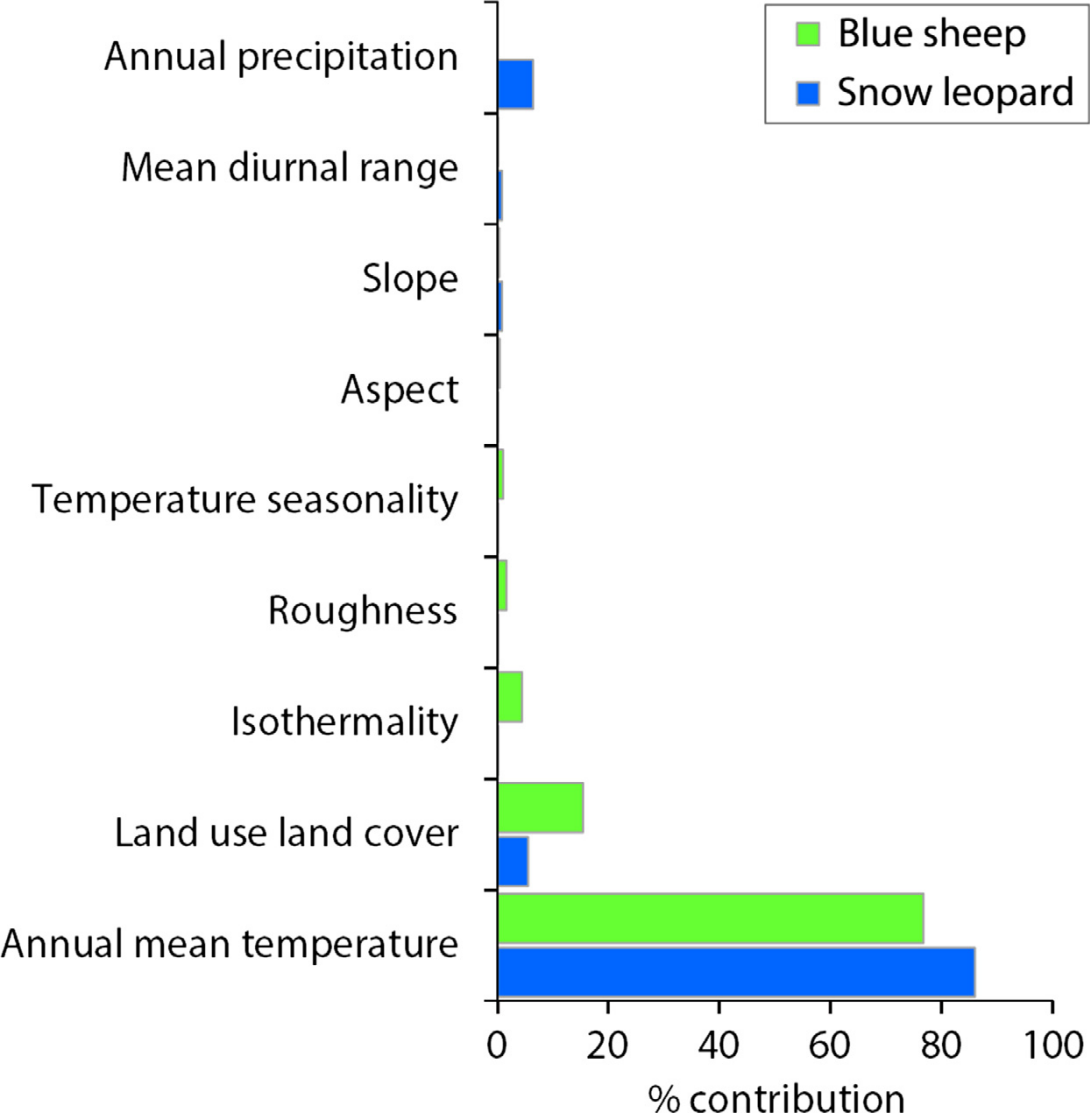
Relative importance of predictor variables for predicted distributions of snow leopard and blue sheep.

The model performance of 21 different models for each species evaluated on the basis of AICc values is given in Table 1. The model with spatial filtering and regularization parameter 5 for snow leopards and spatial filtering and regularization parameter 2 for blue sheep exhibited the best performance. Based on the Jackknife estimates, annual mean temperature influences potential habitats of snow leopard contributing 85.95% to the model (Fig. 1). Likewise, annual precipitation and land cover have the second (6.47%) and third (5.54%) highest contributions to the niche of snow leopard. Similarly, the blue sheep niche is highly influenced by annual mean temperature with a major contribution of 76.68%, followed by land cover (15.39%) and isothermality (4.40%).

**Table 1:** Comparative performance of MaxEnt models in predicting species distribution of snow leopard and blue sheep

Modeling scenarios	Regularization multiplier	Snow leopard					Blue sheep				
		Mean AIC (SD)	Mean AICc (SD)	Mean BIC (SD)	Training AUC	Test AUC	Mean AIC (SD)	Mean AICc (SD)	Mean BIC (SD)	Training AUC	Test AUC
Normal	0.5	7710 (12)	7768 (20)	8054 (35)	0.94	0.94	4327 (19)	4414 (48)	4566 (53)	0.95	0.93
	1	7720 (16)	7750 (21)	7978 (39)	0.94	0.93	4311 (11)	4341 (16)	4466 (23)	0.94	0.93
	1.5	7732 (14)	7750 (19)	7931 (42)	0.93	0.93	4313 (7)	4330 (10)	4433 (16)	0.94	0.93
	2	7720 (12)	7727 (14)	7853 (30)	0.93	0.93	4320 (12)	4331 (15)	4419 (24)	0.94	0.92
	3	7717 (10)	7720 (10)	7801 (13)	0.93	0.93	4333 (9)	4337 (11)	4399 (19)	0.93	0.92
	4	7730 (11)	7732 (11)	7802 (15)	0.93	0.93	4353 (9)	4356 (10)	4406 (17)	0.92	0.91
	5	7742 (11)	7744 (12)	7808 (15)	0.93	0.93	4361 (6)	4362 (6)	4398 (8)	0.91	0.91
Bias	0.5	7860 (17)	7918 (22)	8206 (33)	0.93	0.92	4457 (22)	4548 (48)	4702 (49)	0.93	0.91
	1	7851 (20)	7876 (23)	8086 (35)	0.93	0.92	4425 (18)	4444 (22)	4552 (29)	0.92	0.90
	1.5	7847 (20)	7859 (23)	8017 (39)	0.92	0.91	4428 (19)	4439 (24)	4524 (40)	0.91	0.90
	2	7852 (21)	7860 (22)	7994 (33)	0.92	0.91	4433 (14)	4440 (16)	4513 (26)	0.90	0.89
	3	7855 (19)	7859 (20)	7951 (27)	0.92	0.91	4431 (15)	4435 (17)	4489 (29)	0.90	0.88
	4	7860 (17)	7862 (17)	7929 (19)	0.92	0.91	4424 (12)	4426 (13)	4460 (17)	0.89	0.88
	5	7873 (15)	7874 (15)	7932 (17)	0.91	0.91	4425 (12)	4426 (12)	4455 (15)	0.89	0.88
Spatial filtering	0.5	3778 (18)	3906 (58)	4017 (45)	0.94	0.91	2955 (12)	3054 (33)	3130 (24)	0.95	0.92
	1	3735 (9)	3763 (14)	3867 (19)	0.93	0.91	2933 (11)	2963 (21)	3041 (28)	0.94	0.92
	1.5	3724 (7)	3738 (11)	3820 (18)	0.92	0.91	2934 (8)	2951 (13)	3019 (20)	0.94	0.92
	2	3716 (9)	3723 (11)	3787 (20)	0.92	0.91	2934 (8)	2943 (11)	2998 (18)	0.94	0.92
	3	3709 (5)	3713 (6)	3758 (10)	0.92	0.91	2943 (4)	2947 (4)	2985 (8)	0.92	0.91
	4	3708 (4)	3710 (4)	3747 (9)	0.92	0.91	2950 (5)	2952 (5)	2982 (7)	0.92	0.90
	5	3706 (3)	3708 (4)	3739 (9)	0.92	0.91	2957 (5)	2959 (5)	2988 (6)	0.91	0.90

MaxEnt models were run after 10‐fold cross‐validation, and AUC value shown is the average. Models were evaluated using AICc.

### Current distribution of snow leopard and blue sheep

**Figure 2:**
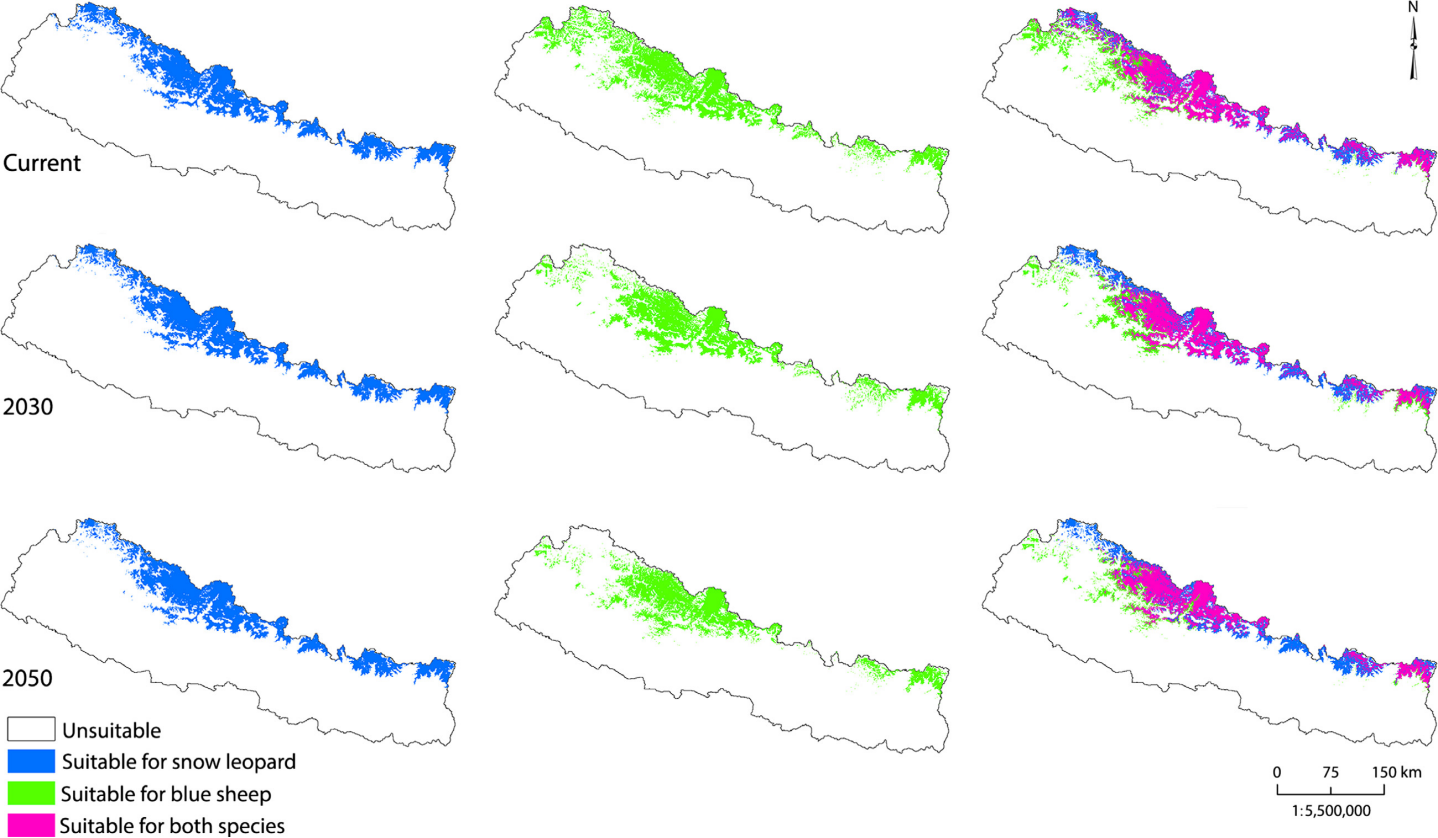
Predicted suitable habitats for snow leopard and blue sheep under different climatic conditions.

The estimated areas of predicted habitats of snow leopard and blue sheep based on the climate‐only models are given in the Table 2. The predicted distribution of snow leopard habitat covers 22,625.34 km^2^ (15.32%) of Nepal. Currently, about 65.98% (14,927.25 km^2^) of the total suitable habitat of the snow leopard falls inside the protected areas with the largest suitable habitat for snow leopard occurring in Annapurna Conservation Area (5183.65 km^2^), followed by Shey Phoksundo National Park including the buffer zone (3235.53 km^2^) and Kanchenjunga Conservation Area (1344.05 km^2^) (Fig. 2).

**Table 2:** Estimated areas (km^2^) of the predicted habitat of snow leopard and blue sheep

Climate scenarios	Area of suitable habitat for snow leopard (% of the total area of Nepal)		Area of suitable habitat for blue sheep (% of the total area of Nepal)
	Without blue sheep habitat	With blue sheep habitat	
Current	22625.34 (15.32)	17190.24 (11.64)	23529.17 (15.93)
2030	22177.57 (15.02)	14685.63 (9.94)	19810.38 (13.41)
2050	21765.30 (14.74)	13482.78 (9.13)	17475.66 (11.83)

Similarly, predicted habitats of blue sheep cover 23,529.17 km^2^ (15.93%) of Nepal primarily in the Annapurna Conservation Area (4945.80 km^2^), Shey Phoksundo National Park including the buffer zone (3909.07 km^2^), Kanchenjunga Conservation Area (1205.87 km^2^), and Manaslu Conservation Area (869.10 km^2^). Altogether, 59.11% (13,909.40 km^2^) of blue sheep predicted habitat falls inside protected areas in Nepal (Fig. 2).

### Change in the predicted habitats of snow leopard and blue sheep

The potential area of suitable habitat for the snow leopard is projected to continuously decline with future climate change from its current distribution by 2030 and 2050 under the RCP4.5 scenario. Model predicts that about 448 km^2^ (1.98%) and 860.04 km^2^ (3.80%) of the potential habitat of snow leopard will be lost by 2030 and 2050, respectively (Table 2). Geographically, the maximum reduction occurs in the Humla District, followed by the Gorkha and Rasuwa districts, whereas suitable habitat is predicted to increase in Dolpa, Mustang, and Manang districts. Fortunately, Snow leopard habitat seems to increase inside the protected areas but decrease outside.

Our model shows that the potential habitat of blue sheep is also reduced with future climate change. The total suitable habitat of blue sheep is restricted to 13.41% in 2030 and 11.83% in 2050 from current potential habitat area of 15.93% of the total area of Nepal which means reductions of 15.8% of current blue sheep habitat by 2030 and 25.72% by 2050 (Table 2). Blue sheep's potential habitat inside protected areas will be reduced under future climate change. About 40.08% of the current protected areas is suitable for blue sheep, and the suitability inside protected areas will be reduced to 36.24% by 2030 and 35.37% by 2050.

### Niche overlap

When we incorporate distribution information of prey in the model, the predicted suitable areas for snow leopard is reduced from the snow leopard climate‐only model under all climatic scenarios: present conditions, 2030, and 2050. Currently, only 11.64% (17,190.24 km^2^) of Nepal remains suitable for the snow leopard, a loss of 24.02% (5435.09 km^2^) in snow leopard habitat after incorporating the predicted distribution of blue sheep. The predicted habitat of snow leopard reduces by 14.57% in 2030 and by 21.57% in 2050 when blue sheep habitat is included compared to 1.98% reduction in 2030 and 3.80% reduction in 2050 based on the climate‐only model.

**Figure 3:**
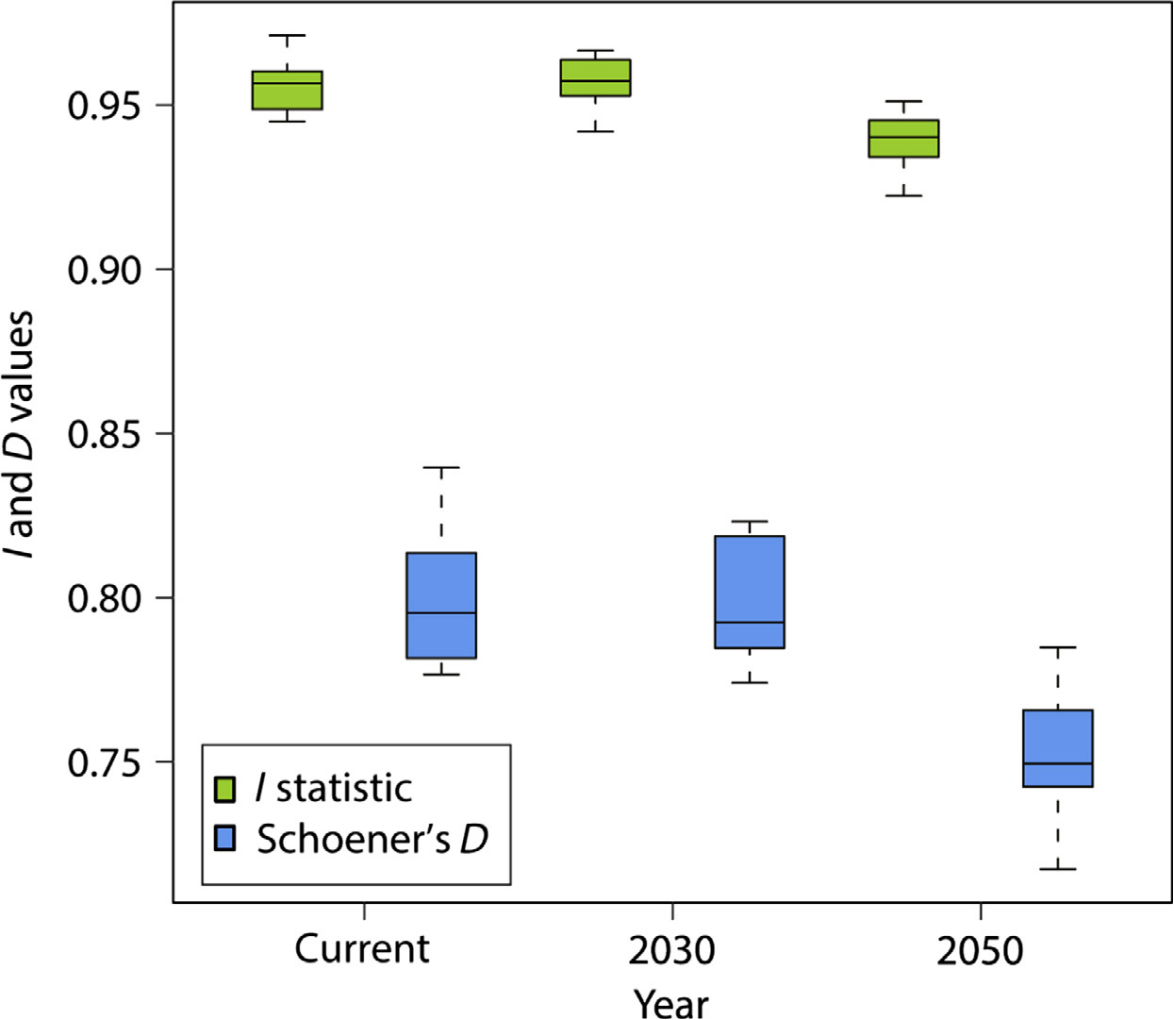
Change in niche overlap between snow leopard and blue sheep under different climatic conditions.

Future climate change may lead to a mismatch in the niche of predator (snow leopard) and prey (blue sheep). The mean Schoener's *D* index value of 0.809 for present climate indicates a high level of overlap between the niches of snow leopard and blue sheep. However, the average *D* value is predicted to decrease to 0.806 in 2030 and 0.764 in 2050 indicating a lower degree of overlap and higher degree of mismatch (Fig. 3). ANOVA test shows that the mean of Schoener's *D* values of ten replicate models for three time periods are significantly different (*F* (2, 27) = 19.283, *P* ≤ 0.0005). The *I*‐statistic values for the niche overlap that is solely based on the probability distribution are also significantly different (*F* (2, 27) = 15.063, *P* ≤ 0.005) (Fig. 3).

## Discussion

We modeled and mapped the distribution of snow leopards and blue sheep under current and future climates by including biological interactions previously overlooked and found spatial incongruity between the predicted distributions of snow leopard and blue sheep. Although MaxEnt is a very popular species distribution modeling approach, a number of recent studies have pointed out the limitations of this approach and suggested species‐specific tuning for the default settings of MaxEnt to improve model performance (Anderson and Gonzalez 2011; Elith et al. 2011; Warren and Seifert 2011; Merow et al. 2013; Muscarella et al. 2014; Radosavljevic and Anderson 2014). Here, we have corrected sampling biases, calibrated default settings of MaxEnt, and evaluated the resulting models based on robust evaluation statistics to overcome these limitations and obtain the best performing model for the species studied. Our results also confirm that spatial filtering of occurrence datasets reduces overfitting (Kramer‐Schadt et al. 2013; Boria et al. 2014) and that the selection of an intermediate regularization multiplier produces the best performing model (Anderson and Gonzalez 2011). Spatial filtering reduced the AICc value significantly compared to the other two scenarios, normal (without spatial filtering and bias corrected) and bias corrected, thus increasing the predictive performance of the model. Furthermore, the AUC values were greater than 0.9 in the selected models, which is recognized as an excellent model (Phillips et al. 2006).

The highest percentage contribution of annual mean temperature to the model suggests that temperature is the most important variable for both snow leopard and blue sheep distributions in Nepal Himalaya. Previous studies (Jackson and Ahlborn 1984; Forrest et al. 2012; Aryal et al. 2014a,b) acknowledged altitude as one of the major limiting factors for snow leopard niche; however, altitude is a surrogate for temperature in Nepal Himalaya: Temperature decreases by 6.2°C with each increase of 1000 m in altitude (La Sorte and Jetz 2010). In fact, altitude does not have a direct impact on habitat suitability, and it indirectly influences distribution through temperature. Furthermore, the inclusion of altitude as a predictor variable in species distribution modeling of mammals negatively affects the predictive power of SDMs (Hof et al. 2012). Likewise, land cover is another important contributor to the predicted habitat of the snow leopard. The predicted habitat of the snow leopard falls mainly in three land cover categories: grassland, snow and ice, and sparse vegetation (Jackson and Ahlborn 1984; Forrest et al. 2012; Aryal et al. 2014b,c).

Based on our model, the estimated area for snow leopard habitat is 22,625 km^2^ in Nepal. Forrest et al. (2012) estimated the area of snow leopard habitat to be 20,000 km^2^ for Nepal in their study over a greater extent of the Himalaya. Our model predicted about 13.12% (2,625 km^2^) more area than that calculated by Forrest et al. (2012). However, these figures are comparable, and the source of the difference might have come from the use of different modeling approaches, resolution of data, bioclimatic variables, and the cutoff points to change the continuous data into binary format of suitable and unsuitable habitats. Forrest et al. (2012) included only patch sizes greater than 500 km^2^ as good snow leopard habitat, whereas we did not exclude habitats based on patch size; this might cause discrepancies in the total estimated areas suitable for snow leopard habitat. However, predicted areas of the suitable habitat for snow leopard decreased significantly after including the predicted distribution of blue sheep in the model. As availability of food fundamentally dictates a species' distribution and abundance, it is not surprising that the predicted niche of a predator is limited by its prey distribution.

Our results show that the existing protected areas of Nepal incorporate significant portions of the predicted habitats of snow leopard and blue sheep. This is because current protected areas of Nepal are highly skewed toward the high‐mountain areas, which comprise 69% of the total protected areas (Shrestha et al. 2010) and are the habitats of both species. For future extension of protected areas to incorporate more snow leopard and blue sheep habitats, if needed, it would be prudent to extend these in the corridors between current protected areas. While current protected areas are sufficient to cover the spatial areas of snow leopard and blue sheep habitats, they cannot guarantee effective habitat conservation as many factors account for effective conservation: prey density, anthropogenic pressures, habitat quality, and human–wildlife conflict (Aryal et al. 2014c). Besides creation of protected areas, threat mitigation measures might be an effective conservation strategy (Hayward 2011). The reported causes of population decline of snow leopard in Nepal are poaching, habitat destruction, retaliatory killing, reduced prey density, and weak enforcement of conservation policies (Aryal et al. 2014b,c): These are likely to be aggravated under future climate change situations. Our analysis indicates that the maximum loss of habitats is predicted to occur outside the current protected areas; therefore, to adapt to future climate change, either new conservation areas need to be established or current conservation areas expanded to cover the predicted loss of habitats under climate change scenario.

Our results reaffirm that predicted habitat of predators declines substantially when prey information is added to the climate‐only model (Peers et al. 2014). Our finding that climate change will lead to a spatial mismatch of snow leopard requirements and blue sheep availability based on the decline in Schoener's D value from current climate to 2030 to 2050 suggesting a lower degree of overlap and higher degree of mismatch. The role of climate in affecting predators through its impact on the relative timing of food requirements and food availability can have a significant effect (e.g., Durant et al. 2007; Broitman et al. 2008). Factors affecting species distribution and predator and prey relationships interact in complex ways (Moritz et al. 2008) and variations in the rates of range shifts among and within species due to differential dispersal abilities affecting a mismatch of predator and prey requirements (Durant et al. 2007; Peers et al. 2014). This mismatch is accentuated in simple ecosystems such as in the Himalaya and with specialist species like the snow leopard.

Evolutionarily, the snow leopard, an example of a stenospecies, could have been driven to adapt to a life in marginal habitats with harsh climatic conditions and low resource availability, making this specialized species particularly sensitive to environmental changes (Lovari et al. 2013). As climate change squeezes the snow leopard to a narrow range between the forest – an unsuitable habitat for this species – and the higher, barren rocky areas, the species marginal habitat (Forrest et al. 2012), snow leopard's distribution will be increasingly restricted with undesirable effects on the conservation of this endangered large cat. The spatial mismatch between blue sheep and snow leopard may also mean snow leopard will have to broaden its diet breadth overlapping its requirement with that of common leopard (Lovari et al. 2013) which is moving toward higher altitude. Competition with this superior competitor for diet and space would be deleterious to the snow leopard. It is interesting to see how other prey species (Himalayan thar, argali, serow, goral, musk deer, marmots) of snow leopard will respond to climate change and influence its distribution given the complexities of incorporating prey information into the climate‐only predictions. Studies included multiple prey species showed that dietary switching occurs in predator species with narrow niches (Peers et al. 2014).

While this study incorporates biotic interactions and addresses key methodological issues of MaxEnt modeling and adds value to previous research in species distribution modeling emphasizing the importance of biotic interactions, it has some limitations. We used the MaxEnt modeling approach considering its popularity and performance, selected a single global climate model based on its predictive accuracy in the study area, and incorporated the distribution of the commonest prey species of snow leopard. Efforts of using multiple distribution models, other global climate models (GCMs), and alternative emission scenarios and incorporating several alternate prey species such as Himalayan thar, argali, serow, goral, musk dear, and marmots might add values to the current study. Therefore, this study should be evaluated on the basis of the limitations of the modeling methods and the availability and quality of the available data given the context of a weak spatial data infrastructure in the Himalaya. Moving beyond these constraints, future studies could be improved by including human pressures, abundance of prey populations, and predicted changes in land cover in the model.

## Conflict of Interest

None declared.

## Supporting information


**Table S1.** Appendix 1: Correlation matrix of topographic, bioclimatic, and other variables.
