# Supplementary material for: Predicting the distributions of predator (snow leopard) and prey (blue sheep) under climate change in the Himalaya
Source: Ecol Evol. 2016 May 18;6(12):4065–75. doi: 10.1002/ece3.2196 (PMC4875782; doi:10.1002/ece3.2196)
Supplement: Supplementary file 1 — Table S1. Appendix 1: Correlation matrix of topographic, bioclimatic, and other variables. [file ECE3-6-4065-s001.doc]

**Supporting information 1**; Correlation matrix of topographic, bioclimatic, and other variables

|  | | | | | | | | | | | | | | | | | | | | | | | | |
| --- | --- | --- | --- | --- | --- | --- | --- | --- | --- | --- | --- | --- | --- | --- | --- | --- | --- | --- | --- | --- | --- | --- | --- | --- |
|  | BIO1 | BIO2 | BIO3 | BIO4 | BIO5 | BIO6 | BIO7 | BIO8 | BIO9 | BIO10 | BIO11 | BIO12 | BIO13 | BIO14 | BIO15 | BIO16 | BIO17 | BIO18 | BIO19 | Altitude | Aspect | Slope | Roughness |  |
| BIO1 | 1.00 | -0.24 | 0.25 | -0.56 | 0.98 | 0.95 | -0.45 | 0.99 | 0.93 | 0.99 | 0.99 | 0.46 | 0.39 | 0.36 | -0.10 | 0.42 | 0.05 | 0.42 | 0.09 | -0.92 | 0.09 | -0.10 | 0.01 |  |
| BIO2 | -0.44 | 1.00 | 0.57 | 0.57 | -0.18 | -0.53 | 0.88 | -0.17 | -0.52 | -0.18 | -0.31 | -0.44 | -0.13 | -0.82 | 0.78 | -0.16 | -0.89 | -0.11 | -0.87 | 0.19 | 0.03 | 0.23 | 0.29 |  |
| BIO3 | -0.20 | 0.58 | 1.00 | -0.31 | 0.18 | 0.08 | 0.12 | 0.24 | 0.05 | 0.21 | 0.26 | -0.08 | 0.15 | -0.59 | 0.56 | 0.15 | -0.75 | 0.21 | -0.76 | -0.35 | -0.05 | 0.26 | 0.38 |  |
| BIO4 | -0.45 | 0.75 | -0.09 | 1.00 | -0.42 | -0.71 | 0.88 | -0.46 | -0.67 | -0.45 | -0.64 | -0.41 | -0.28 | -0.33 | 0.34 | -0.32 | -0.26 | -0.32 | -0.23 | 0.58 | 0.06 | 0.00 | -0.03 |  |
| BIO5 | 0.99 | -0.37 | -0.23 | -0.35 | 1.00 | 0.91 | -0.33 | 0.99 | 0.91 | 0.99 | 0.96 | 0.36 | 0.29 | 0.33 | -0.13 | 0.32 | 0.06 | 0.32 | 0.11 | -0.89 | 0.12 | -0.14 | -0.05 |  |
| BIO6 | 0.95 | -0.67 | -0.28 | -0.65 | 0.92 | 1.00 | -0.69 | 0.91 | 0.99 | 0.91 | 0.97 | 0.50 | 0.33 | 0.54 | -0.37 | 0.37 | 0.33 | 0.36 | 0.36 | -0.87 | 0.07 | -0.16 | -0.10 |  |
| BIO7 | -0.45 | 0.94 | 0.27 | 0.93 | -0.36 | -0.69 | 1.00 | -0.35 | -0.66 | -0.35 | -0.53 | -0.50 | -0.25 | -0.65 | 0.62 | -0.29 | -0.65 | -0.26 | -0.62 | 0.43 | 0.05 | 0.13 | 0.13 |  |
| BIO8 | 0.99 | -0.34 | -0.22 | -0.31 | 1.00 | 0.91 | -0.32 | 1.00 | 0.90 | 1.00 | 0.97 | 0.45 | 0.40 | 0.34 | -0.04 | 0.43 | -0.01 | 0.44 | 0.04 | -0.91 | 0.11 | -0.10 | 0.02 |  |
| BIO9 | 0.96 | -0.63 | -0.24 | -0.63 | 0.93 | 0.99 | -0.65 | 0.92 | 1.00 | 0.91 | 0.95 | 0.42 | 0.24 | 0.51 | -0.43 | 0.28 | 0.33 | 0.27 | 0.36 | -0.85 | 0.08 | -0.19 | -0.14 |  |
| BIO10 | 0.99 | -0.35 | -0.23 | -0.32 | 1.00 | 0.91 | -0.34 | 1.00 | 0.92 | 1.00 | 0.97 | 0.43 | 0.38 | 0.35 | -0.07 | 0.40 | 0.02 | 0.41 | 0.07 | -0.91 | 0.11 | -0.11 | 0.00 |  |
| BIO11 | 0.99 | -0.51 | -0.18 | -0.54 | 0.98 | 0.98 | -0.54 | 0.97 | 0.98 | 0.97 | 1.00 | 0.48 | 0.39 | 0.39 | -0.15 | 0.43 | 0.09 | 0.43 | 0.12 | -0.92 | 0.08 | -0.10 | 0.00 |  |
| BIO12 | 0.43 | -0.69 | -0.87 | -0.17 | 0.43 | 0.52 | -0.46 | 0.43 | 0.46 | 0.44 | 0.43 | 1.00 | 0.94 | 0.75 | 0.12 | 0.95 | 0.34 | 0.93 | 0.32 | -0.42 | -0.03 | 0.03 | 0.15 |  |
| BIO13 | 0.34 | -0.46 | -0.85 | 0.09 | 0.35 | 0.35 | -0.19 | 0.37 | 0.31 | 0.38 | 0.31 | 0.95 | 1.00 | 0.51 | 0.44 | 1.00 | 0.01 | 1.00 | -0.01 | -0.37 | -0.04 | 0.13 | 0.30 |  |
| BIO14 | 0.56 | -0.80 | -0.85 | -0.30 | 0.55 | 0.67 | -0.59 | 0.54 | 0.62 | 0.55 | 0.57 | 0.94 | 0.84 | 1.00 | -0.45 | 0.53 | 0.79 | 0.48 | 0.78 | -0.27 | -0.01 | -0.19 | -0.19 |  |
| BIO15 | -0.35 | 0.80 | 0.12 | 0.85 | -0.29 | -0.59 | 0.90 | -0.24 | -0.56 | -0.25 | -0.44 | -0.20 | 0.11 | -0.39 | 1.00 | 0.41 | -0.79 | 0.46 | -0.80 | 0.08 | -0.01 | 0.33 | 0.47 |  |
| BIO16 | 0.37 | -0.51 | -0.86 | 0.05 | 0.38 | 0.40 | -0.24 | 0.39 | 0.35 | 0.40 | 0.35 | 0.96 | 1.00 | 0.86 | 0.05 | 1.00 | 0.03 | 1.00 | 0.01 | -0.40 | -0.04 | 0.13 | 0.29 |  |
| BIO17 | 0.40 | -0.96 | -0.73 | -0.58 | 0.36 | 0.61 | -0.82 | 0.32 | 0.55 | 0.34 | 0.45 | 0.84 | 0.65 | 0.88 | -0.66 | 0.68 | 1.00 | -0.03 | 0.99 | 0.02 | -0.01 | -0.24 | -0.36 |  |
| BIO18 | 0.35 | -0.45 | -0.84 | 0.09 | 0.37 | 0.37 | -0.18 | 0.39 | 0.32 | 0.39 | 0.33 | 0.95 | 1.00 | 0.83 | 0.11 | 1.00 | 0.64 | 1.00 | -0.06 | -0.41 | -0.04 | 0.14 | 0.32 |  |
| BIO19 | 0.42 | -0.94 | -0.76 | -0.52 | 0.39 | 0.62 | -0.79 | 0.36 | 0.56 | 0.37 | 0.47 | 0.86 | 0.66 | 0.90 | -0.65 | 0.70 | 0.99 | 0.65 | 1.00 | -0.02 | 0.01 | -0.27 | -0.38 |  |
| Altitude | -0.90 | 0.31 | -0.06 | 0.49 | -0.88 | -0.85 | 0.41 | -0.88 | -0.87 | -0.88 | -0.90 | -0.19 | -0.09 | -0.34 | 0.38 | -0.11 | -0.24 | -0.10 | -0.24 | 1.00 | -0.05 | 0.08 | -0.06 |  |
| Aspect | 0.13 | 0.00 | -0.06 | 0.03 | 0.14 | 0.10 | 0.02 | 0.14 | 0.10 | 0.14 | 0.12 | 0.08 | 0.09 | 0.08 | 0.04 | 0.09 | 0.02 | 0.10 | 0.03 | -0.11 | 1.00 | -0.01 | -0.05 |  |
| Slope | -0.06 | 0.26 | 0.26 | 0.08 | -0.06 | -0.12 | 0.19 | -0.05 | -0.11 | -0.05 | -0.07 | -0.19 | -0.14 | -0.23 | 0.20 | -0.16 | -0.22 | -0.14 | -0.25 | -0.05 | -0.02 | 1.00 | 0.43 |  |
| Roughness | -0.13 | 0.21 | 0.22 | 0.08 | -0.15 | -0.18 | 0.16 | -0.13 | -0.17 | -0.13 | -0.14 | -0.16 | -0.11 | -0.20 | 0.18 | -0.13 | -0.21 | -0.11 | -0.24 | 0.06 | -0.07 | 0.45 | 1.00 |  |
| non shaded values for snow leopard, shaded values for blue sheep | | | | | | | | | | | | | | | | | | | | | | | | |
